# The Impact of a Flipped Classroom on the Creativity of Students in a Cake Decorating Art Club

**DOI:** 10.3389/fpsyg.2020.533187

**Published:** 2020-12-29

**Authors:** Li-Chu Tien, Shih-Yen Lin, Hsiang Yin, Jen-Chia Chang

**Affiliations:** ^1^Department of International Trade, Overseas Chinese University, Taichung City, Taiwan; ^2^Department of Tourism, Leisure and Hospitality Management, National Chi Nan University, Nantou County, Taiwan; ^3^Department of Foodservice Management, Vanung University, Taoyuan City, Taiwan; ^4^Graduate Institute of Technological and Vocational Education, National Taipei University of Technology, Taipei City, Taiwan

**Keywords:** flipped classroom, creativity, learning strategies, learning motivation, experimental teaching, creative thinking 2, learning satisfaction, cake decorating art club

## Abstract

This study explored the effect of learning strategies in a student organization on cake art creativity. The participants were 27 student members of a cake decorating art club from one central university in Taiwan. A quasi-experimental pretest-posttest design was adopted, with 90 h of experimental teaching over 16 weeks. The results, which included the use of a questionnaire, classroom observation, and in-depth interviews, suggest that in terms of creativity, the group participating in flipped classroom learning significantly outperformed the group using traditional learning strategies. Furthermore, flipped classroom learning promoted learner motivation and satisfaction.

## Introduction

Decorated cakes play a prominent role in events such as birthdays, wedding anniversaries, and Mother’s Day. Because some consumers demand varied cake decoration, and in response to an increasingly competitive environment, cake decorators must develop more sophisticated techniques to meet consumer needs. Previously, employees were subjected to rigid workplace cultures and were taught, in a mentoring system, to strictly abide by traditional techniques and recipes. Thus, ideas to create new products were considerably limited. Therefore, training cake decorators to be creative artists with a breadth of vision, imagination, and creativity is essential (Ruhlman, 2001). Particularly, in the study of creativity, creativity is not only seen as a way of thinking and acting but also as an indication of innovation capability. Some researchers believe that creativity can be cultivated through education or training (Nickerson, 1999; Fasko, 2000; Donnelly, 2004; Steen-Utheim and Foldnes, 2018).

Decorated cake art is the combination of cuisine and art. A course in cake decoration aims to cultivate cultural, artistic, and creative decorators. In addition to knowledge and technologies imparted by classroom teachers, students can make use of club activities with a group of like-minded people in pursuit of common goals and ideals. Student clubs are not subject to time and curriculum constraints, and a group of profession-oriented people with common interests can make use of extracurricular time to discuss, study, and improve their work and enhance their innovation energy. Students in a cake decorating art club must effectively apply learning methods, strategies, materials, and platforms to achieve ideal learning outcomes and pursue optimal creative expression (Clark and Peterson, 1986).

Taiwan’s Fu Jen Catholic University (2014) used the flipped classroom learning strategy to survey 500 teachers in 2012, and the results revealed the following: the satisfaction percentage was 1.88%; 46% of the teachers indicated that satisfaction showed a “very significant improvement;” 2.67% of the teachers indicated that student test scores had improved significantly; and 2.8% of the teachers indicated that the learning motivation of students had improved significantly. A flipped classroom uses online learning materials and audio-visual aids to augment the pre-existing knowledge and skills of students before the lesson is taught. This approach stimulates student interest in learning and enhances student satisfaction with learning. The actual learning activities that students participate in can easily trigger their learning motivation. Thai et al. (2017) compared the flipped classroom approach with other learning strategies and determined that the flipped classroom exhibits higher learning effectiveness than other teaching methods and exerts a positive effect on student learning satisfaction and intrinsic motivation.

At present, “flipped-learning” is popular and is recognized as the best method to stimulate the creative thinking of students (Bergmann and Sams, 2012; Islim, 2018); the core concept of flipped learning is learner centered. When students become the central focus of a lesson, they can choose what to read and can arrange their learning progress according to their level and time. Thus, they can directly participate in activities that can likely enhance their learning motivation (Hathorn and Ingram, 2002), thus obtaining excellent results (Hwang et al., 2011). Accordingly, by using the flipped classroom approach, cake decorating art clubs can have the following advantages. First, following instructional videos, students can learn the production process, make a cake at home, preliminarily experiment with flavors, and design decoration blueprints in advance. Second, for cake making, furnishing, and decoration, if students have a problem, they can re-watch instructional videos on computer networks, smartphones, and other tools; furthermore, students can be tested through immediate self-testing questions to detect their problems. Third, during the process of self-learning, if students are confused, they can return to the classroom for discussion with classmates and thus improve themselves through cooperative learning, opinion sharing, and problem solving. Fourth, in the process of flipped classroom learning, students continue to learn and think, which may trigger their enthusiasm and further stimulate their creative thinking (Chen and Guo, 2012).

Flipped classroom learning has been applied in formal courses in subject areas such as social humanities, medicine, and nature science, with excellent achievements (Akinoglu and Tandogan, 2006; Strayer, 2007; O’Dowd and Aguilar-Roca, 2009; Hwang et al., 2011). However, no study in the field of cooking club learning activities exists, and no study has developed new community mentor teaching and learning strategies to stimulate student creativity. Therefore, this study attempted to apply the flipped classroom model to a cake decorating art club for the purpose of exploring how flipped classroom learning influences artistic creativity in cake decoration. The purposes of this study were as follows:

(1) To determine whether different learning strategies influence artistic creativity in cake decoration.
(2) To investigate whether flipped learning affects student motivation and the degree of learning satisfaction.

## Literature

### Flipped Classroom

#### Meaning

The flipped classroom is a learning model that promotes communication between teachers and students through pre-class self-preparation, classroom teaching, and after-class exercises. It is learner centered, emphasizing student problem-solving and practical operations as well as the cultivation of high-level thinking and creativity (Chang and Hwang, 2018; Zainuddin, 2018), in contrast to traditional lecture-based teaching with the teacher as the “lecture material deliverer” (González-Gómez et al., 2016; Hao, 2016; Sohrabi and Iraj, 2016). The flipped classroom requires students to initiate self-learning activities through online textbooks, audio, and videos before class, which may improve student pre-knowledge and skills, stimulate learning interest and motivation, encourage participation in actual classroom learning activities, and enhance their independent learning ability and interaction with the learning content. This approach can also increase self-initiated learning (Lin and Tsai, 2020). With in-depth exercises and output in class (He et al., 2016; Chang and Hwang, 2018), the strategy of flipped classroom teaching can be used by students in the practice of learning by doing, and doing by learning. The initiative of learning truly rests on the students themselves, which enhances their learning motivation (Thai et al., 2017).

#### Teaching Model

The flipped classroom, proposed by Bergmann and Sams (2012), is also known as the “reversed classroom.” It reverses the traditional learning environment, and students have to prepare a lesson in a flipped classroom. When encountering difficult problems, students can discuss their problem with each other through online cooperative learning; back in the classroom, teachers can guide students to speculate, discuss, and share results. Lo et al. (2018) adopted the flipped classroom education model in microeconomics courses at the University of Miami, and students read specific parts of a workbook, listened to an audio recording, or previewed related slides before class.

Subsequently, instructors gave a 10-min mini-lecture in the classroom, made guided inquiries, and offered guidance directed toward the assigned reading; during the remaining time, students were arranged to engage in research work, experiments, or cooperative learning. This teaching strategy allowed students to complete the learning of basic knowledge and concepts outside class time, which broke the traditional mode of teacher-centered learning. In the traditional learning mode, students listen in the classroom and perform practice activities afterward. Flipped learning is an innovative learning model.

### Impact of Flipped Classroom on Creativity

Class activities are not teacher-centered but rather involve cooperative learning and critical thinking training; interactive activities are designed to solve problems through group work. In the 1990s, Eric Mazur, a physics professor at Harvard University, observed that students only responded to tests and did not utilize knowledge. The professor then asked students to preview the material, and students then reflected on problems encountered during “prep” through the web; afterward, the professor guided students to speculate and discuss and set them on creative thinking tasks in the classroom.

To motivate creativity, flipped classroom activities must have good patterns in order to attain learning goals. The annular flipped classroom constructed by Gerstein (2011) is a four-stage model; the stages are experiential engagement, concept exploration, meaning making, and demonstration and application. A flipped classroom lesson usually begins with participatory learning activities, such as team cooperation, problem-solving exercises, games, and experimental activities. Outside the classroom, students by themselves watch instructional videos, engage with a podcast curriculum, and visit teaching sites; in addition, they participate in online discussions and in other ways to explore the meaning of related concepts (Zhong et al., 2013). Next, the students complete tests, write a podcast, and make reflective audio recordings and videos to complete the construction of their own knowledge. Finally, teams demonstrate learning outcomes and applications through creative, personalized items and presentations.

The flipped classroom can entail two stages: before class and in class. In the first stage, students must watch instructional videos before class and then conduct practice tasks. In the second stage, in class, students must rapidly complete a few tests, followed by problem solving to complete the internalization of knowledge. Finally, they draw a conclusion and provide feedback. The entire learning process is student-centered, with an activity-oriented approach.

Flipped classroom activities allow students to demonstrate creativity and ingenuity, increasing the worth and uniqueness of work. Furthermore, flipped learning can enhance the artistic value of work and let students perceive and compare advantages and shortcomings of their own work and that of others’ work. Students can sum up the characteristics of creative works and thus strengthen their critical thinking and creativity. Hsieh (2003) found that peer assessment may provide students with opportunities to enhance critical thinking and the ability to express appreciation.

Kaufman et al. (2009) argued that creative thinking and critical thinking skills are always interoperable; the first is divergent thinking and the second is convergent thinking, and the two closely cooperate and inter-operate, leading to effective thinking (Zhang, 2013). Adopting flipped learning in student club activities may effectively stimulate thinking and promote creativity.

### Relationship Between the Flipped Classroom and Learning Motivation and Satisfaction

Lin and Lin (2016) observed that flipped classroom learning helps students acquire knowledge and skills more effectively. Teachers can provide relevant textbook content in advance, for example, ingredient functionality, quantity yield, weight per portion, procedures, and an image of the finished product. This approach may allow students to review the content of the course video recorded by a teacher and initiate self-directed learning based on the recipe. It may also help students grasp key points for preview purposes and help them obtain pre-knowledge of the course content. In flipped classroom learning, students can use Facebook, YouTube, computer networks, smartphones, and other social media and electronic devices to watch video instructions at any time. Again, students can ubiquitously learn without being restricted by time and space (Hwang et al., 2010).

Many scholars have noted that the learning interest of learners in flipped classrooms and the integrity of the curriculum design affect student satisfaction with flipped classroom learning. Flipped classroom learning can enhance motivational effectiveness and learning satisfaction (Kunter et al., 2007; Kim et al., 2014; Chen et al., 2016). Lin and Tsai (2020) confirmed that when teachers adopt the aspects of the flipped classroom teaching model, such as providing relevant materials beforehand, this can help students grasp the key points of a chapter. In addition, posing questions before class can strengthen existing professional knowledge and improve student attention. These strategies can significantly improve student knowledge, skills, and learning satisfaction. The learning strategy of the flipped classroom can enhance student interest in learning and learning motivation and can result in learning satisfaction.

### Creativity

Creative personnel training is the most essential education and economic policy for the 21st century (Chen, 2006). In the wave of knowledge-based economy and globalization, creative personnel training has recently become a key goal at all levels of education in Taiwan (Chen et al., 2005; Tang, 2005). Taiwan launched the “White Paper of Creativity Education” in 2002, which concerns five main areas: individuals, schools, society, industry, and culture. Furthermore, “teaching innovation” and “creativity cultivation” are included in teacher evaluations, appointments, and grading indexes. Creativity is encouraged as a selection criterion in entrance examinations at all levels of school. Creativity has become a future trend in education. Creativity refers to innovating knowledge through mental organization and re-composition, such that a new thing is inspired; this force is called “creativity”. Creativity is based on prior knowledge and experience, and students employ their personality traits such as curiosity, imagination, and adventure. They apply learned creating art to create new, strange, and unique products through a flexible and efficient creative process, which portrays their smooth, unique, and sophisticated abilities (Torrance, 1962; Guilford, 1967). Zhang (2013) interprets creativity as “the formation of a new concept of psychological process beyond the existing experience, breaking the limitation of habits in problem situations; and flexible application experience with problem-solving abilities without conventions and restriction.”

To facilitate research into creative thinking, creativity is often considered as including two components: “fluency” and “elasticity.” Fluency is the ability to smoothly and quickly come up with many solutions for a problem. Elasticity usually refers to the ability to find problems and “get hold of” a diverse and unique answer; that is, a diversity of applications can be found for existing things, which is considered an ability to think elastically (Adams et al., 1994). In brief, according to scholarly perspectives, creativity is defined as an ability to think creatively.

### Assessment of Creativity

Generally, the assessment of creativity is explored from the viewpoint of process, person, product, and press/place. Amabile (1983) indicated that whether the process of thinking is creative depends on the ultimate product. Amabile emphasized that a product or an observed reaction is the ultimate proof of creativity. Runco et al. (2000–2001) have pointed out that “creativity is often defined according to the product.” In general, most scholars define creativity from the creation of a “product,” which is relatively able to reach a consensus (Mayer, 1999).

## Materials and Methods

### The Establishment of Flipped Classroom Teaching

**FIGURE 1:**
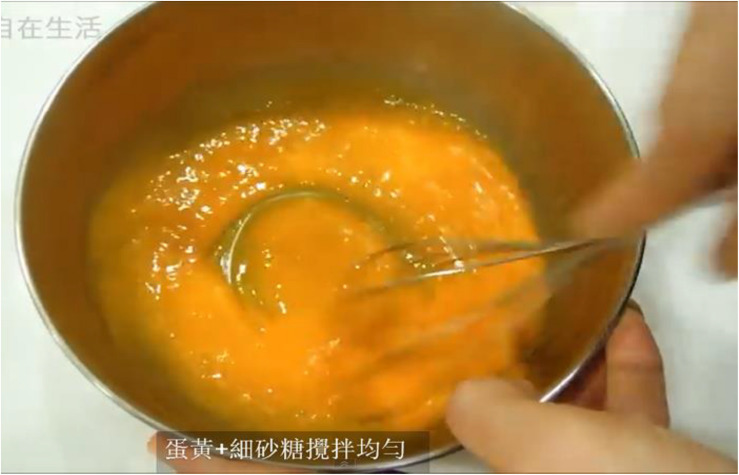
The process of making a chiffon cake.

**FIGURE 2:**
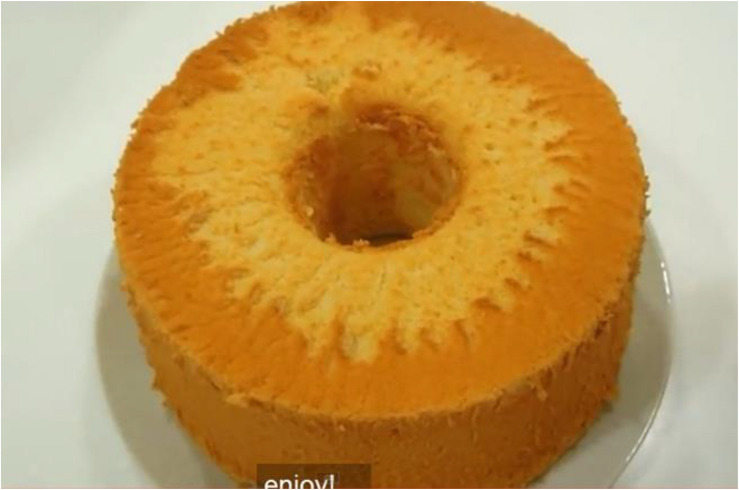
A chiffon cake after baking.

**FIGURE 3:**
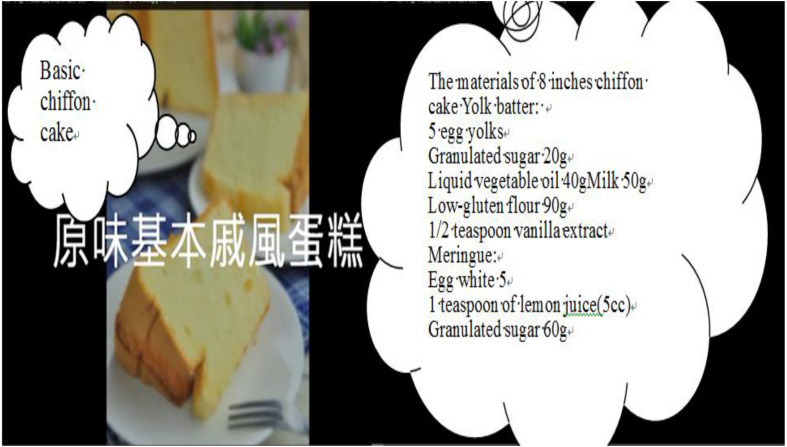
The ingredients of a chiffon cake.

Before the semester began, the construction of a learning platform was completed. Curriculum content (nine units) was placed in smartphones and on Facebook, YouTube, and other sites. Students in the club were then asked to download materials from the platform before the first class. Students could view the content at any time, which offered them flexibility; they were able to learn, no matter when or where (Hwang et al., 2010). Students watched videos to understand materials, such as the production process (as in Figure 1), the baking process, and baking completion (see Figure 2). For example, what are the chiffon cake ingredients? What is the weight of those ingredients? What amounts are required? (see Figure 3).

**FIGURE 4:**
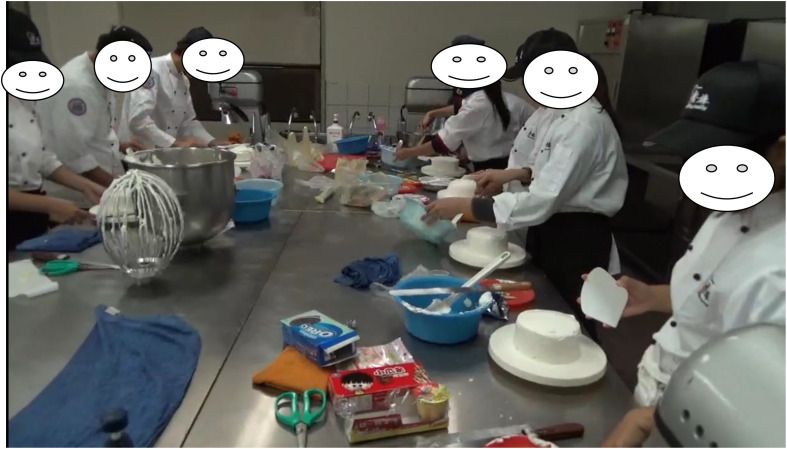
The learning situation of the experimental group.

**FIGURE 5:**
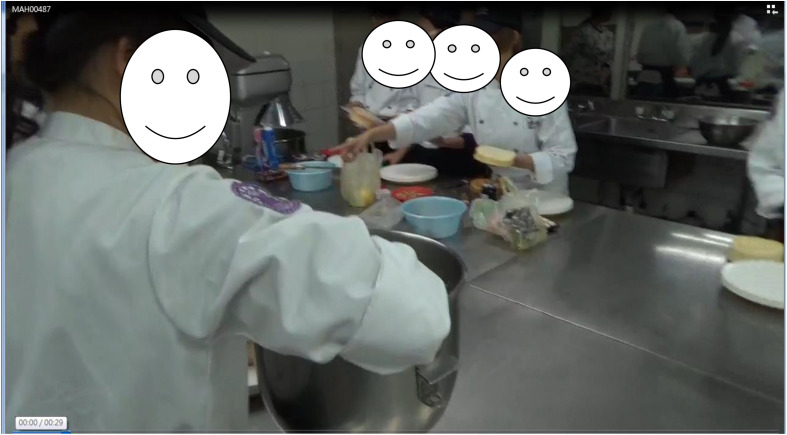
The learning situation of the control group.

Students could re-watch videos if they were puzzled and read and take notes at their own pace. Students that were afraid to ask an instructor a question for the fear of being made fun of and consequently losing interest in learning was thus avoided. In addition, cameras were placed in the classroom to observe learning activities, as shown in Figures 4, 5.

**FIGURE 6:**
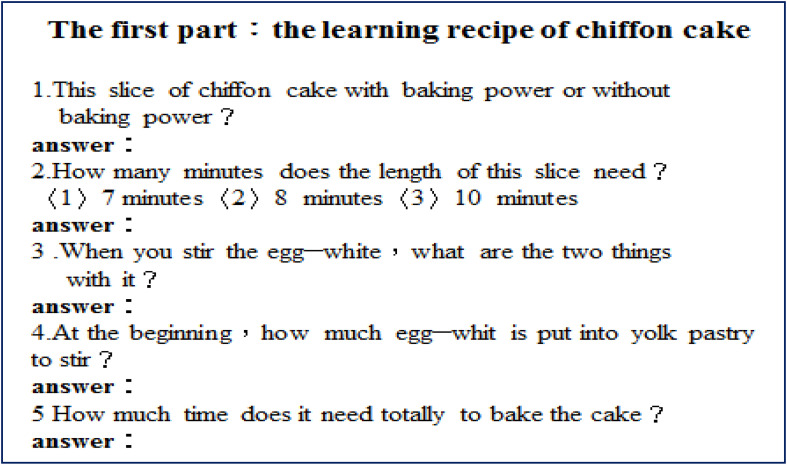
A working sheet of the chiffon cake.

**FIGURE 7:**
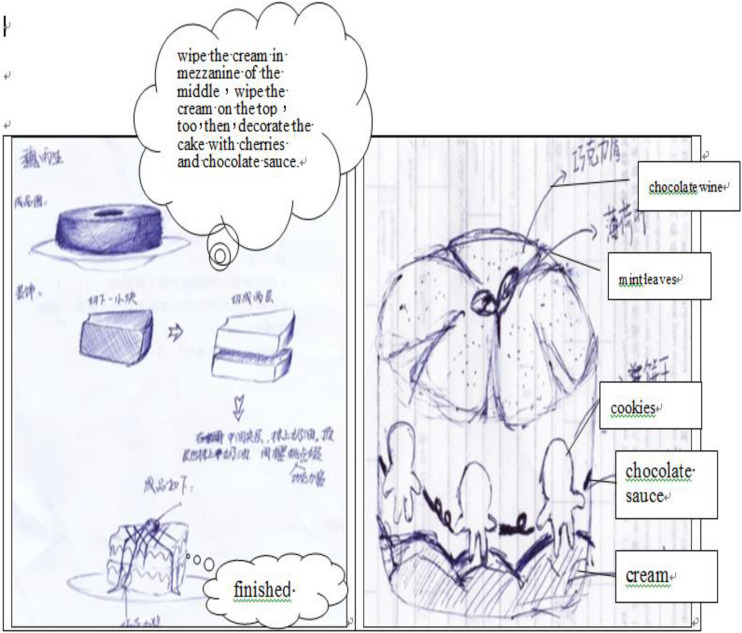
Another working sheet of the chiffon cake.

When students returned to the formal classroom, the instructors inspected learning outcomes and student creativity by means of reviewing worksheets (Figure 6). Instructors thus “got the hang of” what students had learned, any queries students had, and what imagination or creative thinking they had employed. Accordingly, the instructors came to understand the learning situations of the students. They provided guidance by sharing observations, directing activities, moderating discussions, promoting cooperative learning and product presentations and, lastly, encouraging students to create works, as shown in Figure 7.

### Experimental Design and Samples

The experimental course included nine units: chiffon cake, raw cheesecake, yeast muffin, almond cake, brownie cheesecake, cupcake, mousse cake, duo-tone cake, and creative decorated cake.

The experimental procedures of this research refer to Chiou (2009) using the non-equivalent control group design. The experimental subjects were divided into an experimental group and control group.

$$O_{1}X_{1}O_{2}$$

$$O_{1}X_{2}O_{2}$$

Before the experiment, both groups were given the same topic for pre-test (*O*_1_). After the pre-test, the experimental group used the flipped classroom method (*X*_1_); the control group used the traditional narration method (*X*_2_). After the experiment, both groups accepted the post-test on the same topic (*O*_2_) to detect the differences in learning between the two groups of students.

A sample of 27 people (aged approximately 19–20 years) was randomly selected from a cake decorating student club in the hospitality department of a university. For a period of 16 weeks, 5 h per week, 12 people were allocated into the experimental group, which was further divided into four groups; each group of three people participated in flipped classroom learning. The control group of 15 people was divided into five groups, with each group consisting of three people using traditional learning. The number of the control group and marshaling was the same as the experimental group; the two groups were tested separately, and the pre-test score was taken as the covariate variable.

For the sake of objectivity, both the groups had the same instructor, a specialized technical associate professor with approximately 20 years teaching experience and chef experience in a domestic five-star West Point hotel.

### Assessment Tool

#### Matrix for Creative Culinary Works Scale

Matrix for Creative Culinary Works is a creative cooking product rating scale developed by Horng and Lin (2009). This scale was adopted and modified by four experts (two from the hospitality department and two from the industry). It contained four subscales.

The first was for “professional techniques” and contained (1) cake decoration tips, (2) operation correctness of the batter mixing process, (3) cake baking process operation, and (4) production process and hygiene. The second was for “color” and “shape” and measured (1) uniqueness of painting, (2) uniqueness of shape and style, (3) holistic shape, and (4) esthetic appearance. The third subscale was for “flavor” and “taste” and measured (1) internal meticulous organization, (2) uniqueness of flavor, and (3) density and refreshment. The fourth was the overall assessment and measured (1) commerciality, (2) attractiveness, (3) cake named after a creative theme, (4) compatibility with story, and (5) creativity of cake integrity.

This scorecard, with a total of 16 questions, was used for the pre-test and post-test. Scores were given on a 5-point Likert scale from “high” (5 points) to “low” (1 point). The reliability of its items ranged from 0.88 to 0.95, and the reliability of the overall scale was 0.98, indicating good reliability. In addition to on-site instructor scoring, according to the recommendation of Associate Professor Huang’s students, creative ideas and achievements were presented in a photo, tagged with a cake decorating score sheet (Table 1), and it was then sent to experts for rating on the basis of the designed “creative cooking product rating scale.”

**TABLE 1:** The score sheet of cake decoration.

Name of product:	Maker:
Full face photo	Profile photo

Taste: Such as chocolate or fruit The story’s description:	

#### Learning Motivation and Satisfaction Scale

To understand students’ feelings and views on learning motivation and satisfaction regarding learning strategies, the authors referred to the measurement tool compiled by Chiou et al. (2004), the “Learning Attitudes Scale,” which has a Cronbach’s α of 0.89. The survey instrument was reviewed by three experts, which made the learning attitude questionnaire of this study more valid and reliable. In-depth interviews of the experimental group were conducted immediately after the completion of the survey to discuss learning motivation.

### Research Implementation Procedure

The experimental procedure is outlined in Table 2. The implementation was as follows. The teaching experiment was divided into three stages. The first stage was the preparation stage and included (1) setting up a digital teaching platform and a classroom camera; (2) making a cake decoration teaching video, and creating a curriculum plan and a study list for student activities. Two pastry instructors and two baking curriculum experts from inside and outside of the school were invited to review and revise course materials. Therefore, the content of the teaching material accurately corresponded with expert perspectives; and (3) developing the creative cake art product scale, with questions for the pre-test and post-test, to understand the creativity of the two groups of students. The pre-test was conducted with 35 students who had completed a cake decoration course. Reliability and validity were tested, and the items were screened. The second stage was the pre-test stage, which included the following. (1) Instructors used traditional teaching methods to teach three units (e.g., chiffon cake and cheesecake) to both groups of students for a total of 12 h over 4 weeks. (2) One week after the three units were completed, the pre-test (the creative cake art product assessment scale derived from the Matrix for Creative Culinary Works) was administered to examine the creativity of the two groups. The third stage was the formal experimental stage as follows: (1) The experimental group implemented flipped classroom learning methods, whereas traditional learning methods were applied with the control group. The learning content included brownie cheesecake, cupcakes, mousse cake, two-color cake, and creative cake decoration, for a total of five units. The duration of the experimental teaching period was 13 weeks (39 h). (2) One week afterward, both groups took the same creative cake art assessment post-test. Interviews were conducted with the experimental group regarding their learning motivation and teaching and learning activities.

**TABLE 2:** The process of the study implementation.

Stage	Activities
**The first stage** (The 1st week) Preparation phase	(1) Make and select instructional videos of making decorated cakes. (2) Complete experts’ examination of instructional videos. (3). Explain the semester courses and activities, and elucidate the meaning and learning method of the flipped classroom. (4) Describe the content of teaching experimental activities and the precautions to the experimental group. (5) Develop the questions of the “Creative Decorated Cake Product Checklist,” and choose a class of 35 people (baking group) to conduct the pre-test. (6) The preparation of the activities curriculum and worksheets for students in the club. (7) Analyze the pre-test data and prepare the formal “Creative Decorated Cake Product scale.” (8) Build and test the digital teaching platform. (9) Set up a camera in the classrooms to understand the students’ learning situation.
**Second stage** (From week 2 to week 17) Formal experiment stage	(1) Grouping: divided into two groups for the teaching test, the experimental group adopts a flipped classroom teaching strategy; control group takes the traditional teaching strategy. (2) Conducting the pre-test: a pre-test is undertaken by the two groups for measuring the prior knowledge of students. (3) Requests that the experimental group students must watch the teaching video and practice exercises before class; for the previewed videotape, conduct panel discussions, ask questions, conduct cooperative learning, and demonstrate works in the club activities. (4) Set the VCR to watch the students learning, and write a record of the basis for modifying the next curriculum activities. (5) Teachers’ guidance, rethinking, and curriculum adjustment
**The third stage** (Week 18) Stage of completion	(1) A post-test is undertaken by the two groups after the end of the experiment. (2) The questionnaire: to understand the feelings and views of the experimental group students toward the teaching activities. (3) Interview: to record the feelings and views of the experimental group students toward the teaching activities. (4) Data analysis and writing.

### Data Analysis Method

Because the pre-test scores of the experimental class and the control class were different, in order to avoid the difference in the previous levels of the two classes affecting the experimental results, a covariance analysis (ANCOVA) was used to solve this problem. In this study, an ANCOVA was performed using the pre-test as the covariate and the post-test as the dependent variable. Before using the ANCOVA, a within-group regression coefficient homogeneity test was first performed to see if it was appropriate to use this statistical method.

## Results Analysis

### The Effect of Different Learning Strategies on Student Creativity

#### Experiment Participants

This study adopted a quasi-experimental design. Again, the experimental group consisted of 12 students, accounting for 44.4% of the total; the control group consisted of 15 students, accounting for 55.6% of the total. To eliminate interference from unrelated variables, single factor one-way univariate analysis of covariance (ANCOVA) was used to correct for any interfering variables and improve the internal validity of the results. The learning strategies were viewed as independent variables, the pre-test scores were covariates, and the post-test scores were the dependent variable.

#### The Comparison of Two Averages and Standard Deviation

**FIGURE 8:**
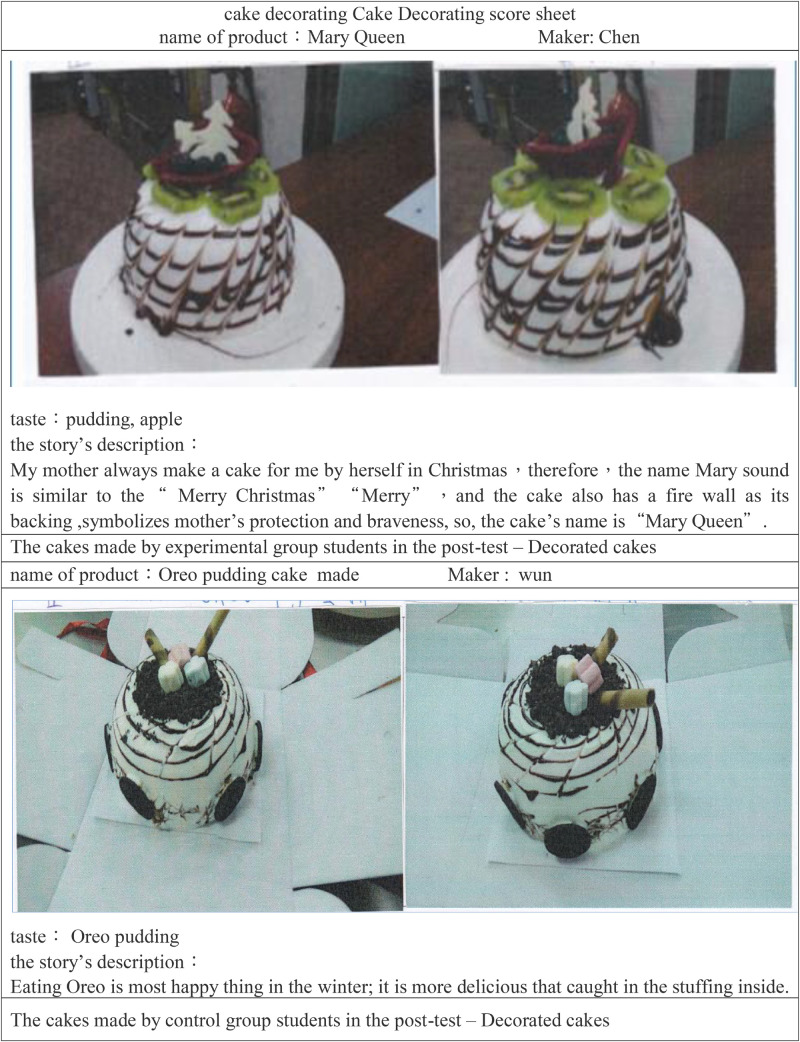
The cakes by the two groups of students in the post-test.

Prior to the experiment, students had knowledge of cake decoration. From the first to the 7th week, the instructor, course content, time, duration, learning hours, worksheets, and teaching methods were identical. The pre-test was performed for the two groups in the 8th week (Figure 8).

The results indicate that the average pre-test score of the experimental group was 43.5 points; the score of the control group was 42.8 points. The difference between the two groups was only 0.7 points, which was insignificant. After the pre-test, the experiment began. The experimental group adopted the flipped learning strategy, whereas the control group made use of traditional learning strategies. The post-test was conducted in the 16th week. The average post-test score for the experimental group was 52.2 points, with a standard deviation of 3.95 points, and that of the control group was 15.7 points, with a standard deviation of 2.06; the difference between the two averages was 6.5 points. Effect size measured the magnitude of the influence of the learning method and was computed as the mean score of the post-test minus the mean score of the pre-test, divided by the standard deviation of the pre-test (Cohen, 1988). According to Cohen’s (1988) rule, the effect size of the experimental group was 0.62, indicating that the use of flipped classroom teaching had a moderate effect on student creativity. The effect size for the control group was 0.06, indicating the use of traditional teaching had no effect on student creativity.

The results therefore indicate that the performance of the experimental group was significantly better than that of the control group. Many studies have determined that teachers’ guidance, thinking, and behavior affect the learning and thinking of students (Barell, 2003; Costa, 2006). Yeh (2004) have affirmed that classroom interaction, learning strategies, and a supportive learning environment can stimulate and enhance student creativity. Clark and Peterson (1986) observed that student thinking, planning, and decision making constitute the major part of the psychological environment of learning; classroom teaching, teaching materials, learning methods, teaching design, and the teacher-student interaction exert a direct effect on learning quality.

#### One-Way Univariate Analysis of Covariance

As Table 3 indicates, the result of the test for homogeneity was *F* = 0.998 (*P* = 0.328, > 0.05), which was not significant. Thus, the homogeneity assumption of the regression coefficient group was not violated, in line with the basic assumption, and an analysis of covariance was conducted. As observed in Table 4, excluding the effect of the pre-test scores (covariance), its *F*-value was 28.717 and the *P*-value (*P* < 0.001) was significant, which implies that the flipped classroom learning strategy was superior to traditional learning strategies.

**TABLE 3:** Descriptive analysis.

Classes	Experimental group		Control group	
	Mean	SD	Mean	SD
Pre-test	43.98	18.45	42.7	19.17
Post-test	52.2	15.7	42.07	16.43
Effect size	0.62		0.06	
N	12		15	

**TABLE 4:** The summary table for the homogeneity test of creativity of different learning strategies.

Source	SS	df	Mean square	*F*	Sig.
Group	4.316	1	4.316	0.526	0.476
Pre-test	29.462	1	29.462	3.591	0.071
Group*pre-test	8.192	1	8.192	0.998	0.328
Error	188.718	23	8.205		
Total	64169.000	27			
Corrected total	512.667	26			

### The Effect of Different Learning Strategies on Learning Motivation and Learning Satisfaction

With the use of a questionnaire, a learning satisfaction survey of the experimental group was conducted. This was followed by in-depth interviews exploring the feelings and views of the experimental group regarding the teaching activities. The analysis results are as follows:

(1) Can flipped classroom learning trigger your motivation?

In all, 83% of the students strongly agreed and 17% agreed; thus, all students agreed that flipped classroom learning strategies, for decorated cake creativity, were able to trigger their motivation. Watching teaching videos at home was helpful for understanding cake ingredients, cake making processes, cake decorating, cake baking, cake painting, and baking time. When students had a basic understanding of making a cake, they had more enthusiasm to work together and discuss cake modeling, painting, or flavoring with classmates when returning to the club. Again, if they became confused, students were able to re-watch videos, which avoids the fear of asking the teacher questions or of being made fun of and consequently losing interest in learning.

(2) Can the flipped teaching strategy make you better understand the baking process of making a decorated cake and the amount of water storage capacity?

In all, 92% of students strongly agreed and 8% agreed. In baking, the amount of water plays a key role in success or failure. Two processes are especially critical to success in the process of making a cake. One is the batter mixing process, and the other is the amount of water storage capacity in the baking process. Only a perfect operation can produce high-quality cakes. Liu (1999) pointed out that stirring is determinable for batter quality in the mixing process, and stirring is a crucial factor in the physical structure of a cake; the degree of agitation is essential to cake quality.

Xu et al. (1996) confirmed that the amount of water affects whether the cake is dense or solid when baked. This professional knowledge and the production process were visualized and presented through a teaching video, and then, the instructor and students discussed them and shared outcomes, which helped students fully understand the skills of cake baking.

(3) Can the flipped classroom learning strategy make learning the art of decorated cake making simpler?

Here, 100% of students agreed. All students agreed that the flipped classroom can make learning easier because the video functions of pause or rewind for repeat viewing can be used with a teaching video in order to understand the cake making process. For example, overlong stirring time leads to large specific gravity, small volume, large holes, stickiness, and bad taste, thus, the cake is not agreeable. However, insufficient mixing time results in sparse batter that is difficult to fully bake; thus, the interior of the product will be wetter.

(4) Can the flipped classroom make you more creative in the process of learning?

In all, 92% of students strongly agreed; 8% agreed. Most students agreed that watching teaching videos before class was helpful for understanding the process of cake making, which enabled more imaginative cake modeling. With worksheets, students drew their own ideas into a single pattern and then returned to the club to discuss them with other students, observe other products, and write the story of their product. The appearance of a decorated cake is created through story, which makes the product richer and more creative.

(5) Can flipped classroom learning make you more creative in the process of making artistic cakes?

Overall, 83% of students strongly agreed, and 17% agreed. Most students stated that, by watching the process of cake making, the brain imagines a wide range of shapes and different flavors of cake. Then, in the club, they can discuss the shape of the cake or the recipe of a unique taste with others. Mutual observation and cooperative learning may contribute to the creation of a good product; the works of decorated cake would thus contain more diversity and creativity. In traditional learning, students only followed instructions given by the instructor or the president of the club to make duplicate cakes, and fewer creative ideas entered the product.

(6) Do you think that the “flipped classroom learning strategy” is suitable for learning creativity in artistic cake design?

All 100% of the students strongly agreed. Most students felt that the flipped classroom was very good for their learning. By watching teaching videos, they were able to grasp the principles and procedures of cake making, for example, the degree of agitation, formulation of additives, capacity of water storage in the baking process, baking, molding, cooling, and decoration. Because they became quite familiar with the cake ingredients and the use of the appliances, they could successfully complete cake production.

## Conclusion and Suggestion

### Conclusion

This study sheds light on the effect of different learning strategies for creativity in the area of cake decoration learning. The results revealed that the experimental group, students undergoing flipped learning, had better performance in creativity than the control group; that is, students undergoing traditional learning. The flipped classroom is a student-centered learning mode; before the course, students were able to watch teaching videos to learn the entire production process of decorated cake baking, including ingredients, mixing, baking, molding, cooling, decorating, and shaping. Furthermore, students painted their ideas and stories on worksheets; the process of drawing may spark the imagination and inspire creativity. One student mentioned, with respect to the appearance and taste of the cake, that they wanted to use extruded whipped cream to make a fairy tale character, and that the formulation should be of delicate taste. When the students decorated a cake at home, they encountered difficulties. Therefore, they not only repeated production but also continued to imagine the end product. Therefore, even when they failed to successfully complete the cake decoration, they generated numerous ideas, which could stimulate more creative works, through the guidance of the instructor and peer discussion, when they returned to the classroom.

Chung and Chow (2004) argued that active learning can inspire creativity, but students must have prior knowledge, life experiences, and a background related to the issue; students can participate in discussions with other students in the classroom or engage in active learning after class (Lee and Wang, 1998). Vygotsky (2004) had the view that creativity is based on the elements of past experiences; this is because the human brain extracts memories and, simultaneously, breaks down, links, and remodels experience to generate new propositions and new behavior (Vygotsky, 1987). Accordingly, by means of flipped classroom learning, students understood the entire process of decorated cake making. They drew what they watched and imagined on worksheets; in the process of drawing, they continued thinking, conceiving, and asking questions. Then, they returned to the club, where they received guidance from the instructor. In addition, through cooperative learning, they learned from each other, which inspired them to employ creativity in creating products with unique shapes or flavors.

In-depth interviews revealed that students believed that flipped classroom learning can increase motivation. In addition to making cake making simpler, flipped classroom learning prompted their own unique ideas. The process of making a decorated cake is cumbersome and complicated. The weight of ingredients, stirring, baking time, foaming, the appearance of whipped cream, and decorating all must be considered. Flipped classroom learning can proceed according to student reading speed; furthermore, students can repeatedly watch teaching videos and make a cake at home in advance. In the production process, students may learn spontaneously and find solutions actively if they encounter difficulties or feel confused. Accordingly, the students confirmed that autonomous flipped learning made cake classes easier to understand, stimulated learning motivation, maintained learning, and allowed them to achieve learning goals.

Classroom observation revealed that most students in the experimental group exhibited more serious learning attitudes and were more attentive, and only a few students played with their phone or chatted. By contrast, students in the control group were “hurry-scurry” and appeared unsure of what to do; some played on their phone alone or chatted with others, and only a few students took serious notes and followed the steps to make a cake. As for the speed of completing cake making, the control group was slower than the experimental group. Classroom observation also suggested that the experimental group outperformed the control group in terms of the student learning ability and the taste and shape of cakes. The results confirmed that the flipped classroom is an effective learning strategy for creativity.

### Suggestion

The results of this study cannot be generalized to students of Chinese or Western cooking, housekeeping services, or customer service technology, or to other student communities. Furthermore, this was a short-term study; a long-term study would be required to ascertain the retention of the identified learning outcomes.

After observing teaching in the classroom, the researchers discovered that the instructor retained traditional teaching methods. The instructor was unable to make sufficient use of technology and IT and thus encourage students to be innovative and capable of problem solving. The flipped classroom teaching mode does not have a fixed pattern; thus, instructors cannot give proper guidance for student creativity and innovation. Accordingly, future research can investigate whether the teacher’s ability to use IT affects student innovation and creativity.

At present, many DIY businesses are attached to physical stores in the baking industry. Thus, consumers without baking experience can use mobile phones to download and follow videos to make a cake and, when watching a video, they can ask the clerk if they have a problem.

Due to the prevalence of online learning, learning is no longer limited to a “classroom”; and learning is not limited by time or place. Future research can explore whether adding music to teaching videos improves student interest, motivation, and concentration.

## Data Availability Statement

The original contributions presented in the study are included in the article/supplementary material, further inquiries can be directed to the corresponding author.

## Ethics Statement

Ethical review and approval was not required for the study on human participants in accordance with the local legislation and institutional requirements. Written informed consent from the (patients/participants or patients/participants legal guardian/next of kin) was not required to participate in this study in accordance with the national legislation and the institutional requirements.

## Author Contributions

L-CT: conceptualization, methodology, software, writing – reviewing, editing and reviewing opinion response, supervision, and validation. S-YL: data curation, investigation, software, writing – original draft preparation, and reviewing opinion response. HY: visualization, writing – editing and English revision and proofreading, and methodology. J-CC: visualization, English revision, and proofreading. All authors contributed to the article and approved the submitted version.

## Conflict of Interest

The authors declare that the research was conducted in the absence of any commercial or financial relationships that could be construed as a potential conflict of interest.

## References

[B1] Adams G. R. Gullotta T. P. Adams C. M. (1994). *Adolescent Life Experience.* California, CA: A division of Wadsworth, Inc.

[B2] Akinoglu O. Tandogan R. (2006). The effects of problem-based active learning in science education on student’s academic achievement, attitude and concept learning. *Eurasia J. Math. Sci. Technol. Educ.* 3 71–81. 10.11648/j.sjedu.20200803.12

[B3] Amabile T. M. (1983). A model of creativity and innovation in organizations. *Res. Organ. Behav.* 10 123–131.

[B4] Barell J. (2003). *Developing more Curious Minds.* Alexandria, VA: Association for Supervision and Curriculum Development.

[B5] Bergmann J. Sams A. (2012). *Why Flipped Classrooms are Here to Stay.* Available online at: http://www.edweek.org/tm/articles/2012/06/12/fp_bergmann_sams.html?tkn=WPCC1Rxu4%2FbCFsj3iEU3%2Bqk97aMS3xc0jkgq&cmp=clp-sb--edtech (accessed 29, October).

[B6] Chang S.-C. Hwang G.-J. (2018). Impacts of an augmented reality-based flipped learning guiding approach on students’ scientific project performance and perceptions. *Comput. Educ.* 125 226–239. 10.1016/j.compedu.2018.06.007

[B7] Chen C. Y. Wu T. W. Chen Z. C. (2005). The development of creativity education in Taiwan: a historical review. *Bul. Educ. Resour. Res.* 30 97–111.

[B8] Chen L. A. (2006). *Creating Teaching Theory and Practice.* Taipei: Psychology Press.

[B9] Chen M.-J. Guo C.-Y. (2012). Post-standardization: an alternative approach toward curriculum evaluation. *J. Curriculum Teach.* 15, 1–24.

[B10] Chen S. C. Yang S. J. H. Hsiao C.-C. (2016). Exploring student perceptions, learning outcome and gender differences in a flipped mathematics course. *Br. J. Educ. Technol.* 47 1096–1112. 10.1111/bjet.12278

[B11] Chiou C. C. (2009). Effects of concept mapping strategy on learning performance in business and economics statistics. *Teach. High. Educ.* 14 55–69. 10.1080/13562510802602582

[B12] Chiou C. C. Huang H. S. Hsieh J. H. (2004). Applying hypermedia assisted concept maps to construct accounting inventory teaching material. *J. Natl. Taip. Teach. Coll.* 17 57–84.

[B13] Chung J. C. C. Chow S. M. K. (2004). Promoting student learning through a student-centred problem-based learning subject curriculum. *Innov. Educ. Teach. Int.* 41 157–168. 10.1080/1470329042000208684

[B14] Clark C. M. Peterson P. L. (1986). “Teachers’ thought process,” in *Research on Teaching*, ed. Wittrock M. C. (New York, NY: Macmillan), 255–296.

[B15] Cohen J. (1988). *Statistical Power Analysis for the Behavioral Sciences.* New Jersey, NJ: Lawrence Erlbaum.

[B16] Costa L. (2006). Five themes in thought-full curriculum. *Think. Skills. Creat.* 1 62–66. 10.1016/j.tsc.2005.03.002

[B17] Donnelly R. (2004). Fostering within an imaginative curriculum in higher education. *Curriculum. J.* 15 155–166. 10.1080/0958517042000226810

[B18] Fasko J. D. (2000). Education and creativity. *Creativity Res. J.* 13 317–327.

[B19] Gerstein (2011). *The Flipped Classroom Models: A Full Picture [EB/OL].* Available online at: http//:usergeneratation.wordpress.com/2011/06/13/the-flipped-classroom-model-a-Full-Picture

[B20] González-Gómez D. Jeong J. S. Airado Rodríguez D. Cañada-Cañada F. (2016). Performance and perception in the flipped learning model: an initial approach to evaluate the effectiveness of a new teaching methodology in a general science classroom. *J. Sci. Educ. Technol.* 25 450–459. 10.1007/s10956-016-9605-9

[B21] Guilford J. P. (1967). *The Nature of Human Intelligence.* New York, NY: McGraw-Hill.

[B22] Hao Y. (2016). Exploring undergraduates’ perspectives and flipped learning readiness in their flipped classrooms. *Comput. Hum. Behav.* 59 82–92. 10.1016/j.chb.2016.01.032

[B23] Hathorn L. Ingram A. (2002). Cooperation and collaboration using computer-mediated communication. *J. Educ. Comput. Res.* 26 325–347. 10.2190/7mkh-qvvn-g4cq-xrdu 22612255

[B24] He W. Holton A. Farkas G. Warschauer M. (2016). The effects of flipped instruction on out-of-class study time, exam performance, and student perceptions. *Learn. Instr.* 45 61–71. 10.1016/j.learninstruc.2016.07.001

[B25] Horng J.-S. Lin L. (2009). The development of a scale for creative culinary products. *Creat. Res. J.* 21 54–63. 10.1080/10400410802633491

[B26] Hsieh C. T. (2003). *Integration Information Into Visual Arts Appreciation Instruction for Elementary Giftedness Class.* Master’s Thesis, Institute of Special Education, National Taiwan Normal University, Taipei.

[B27] Hwang G.-H. Lee L.-M. Wang H.-Y. Hong P.-J. Wu J.-L. Lai X.-L. (2010). Development and effectiveness analysis of ubiquitous learning systems- a case study for elementary school children to recognize campus plants. *Int. J. Digit. Learn. Technol.* 3 19–41.

[B28] Hwang G. J. Wu P. H. Ke H. R. (2011). An interactive concept map approach to supporting mobile learning activities for natural science courses. *Comput. Educ.* 57 2272–2280. 10.1016/j.compedu.2011.06.011

[B29] Islim O. F. (2018). Technology-supported collaborative concept maps in classrooms. *Act. Learn. High. Educ.* 19 131–143. 10.1177/1469787417723231

[B30] Kaufman J. C. Baer J. Cole J. C. (2009). Expertise, domains, and the consensual assessment technique. *J. Creat. Behav.* 43 223–233. 10.1002/j.2162-6057.2009.tb01316.x

[B31] Kim M. K. Kim S. M. Khera O. Getman J. (2014). The experience of three flipped classrooms in an urban university: an exploration of design principles. *Internet High. Educ.* 22 37–50. 10.1016/j.iheduc.2014.04.003

[B32] Kunter M. Baumert J. Köller O. (2007). Effective classroom management and the development of subject-related interest. *Learn. Instr.* 17 494–509. 10.1016/j.learninstruc.2007.09.002

[B33] Lee J. C. Wang C. C. (1998). The instruction of project course through creative problem solving method. *Vocat. Bimonthly* 45 39–44.

[B34] Lin C. L. Tsai C. Y. (2020). The gender difference of learning perception in the flipped teaching of high school technology curriculum. *Chin. J. Sci. Educ.* 28 1–23. 10.6173/CJSE.202003_28(1).0001

[B35] Lin S.-F. Lin H.-S. (2016). Learning nanotechnology with texts and comics: the impacts on students of different achievement levels. *Int. J. Sci. Educ.* 38, 1373–1391. 10.1080/09500693.2016.1191089

[B36] Liu L. H. (1999). *World’s Newest Western-Style Pastry Manufacture Encyclopedia.* Taiwan: Whole wheat baked Press.

[B37] Lo C. K. Lie C. W. Hew K. F. (2018). Applying “first principles of instruction” as a design theory of the flipped classroom: findings from a collective study of four secondary school subjects. *Comput. Educ.* 118 150–165. 10.1016/j.compedu.2017.12.003

[B38] Mayer R. E. (1999). “Fifty years of creativity research,” in *Handbook of Creativity*, ed. Sternberg R. J. (Cambridge, MA: Cambridge University Press), 449–460. 10.1017/cbo9780511807916.024

[B39] Nickerson R. S. (1999). “Enhancing creativity,” in *Handbook of Creativity*, ed. Sternberg R. J. (Cambridge, MA: Cambridge University Press), 392–430. 10.1017/cbo9780511807916.022

[B40] O’Dowd D. K. Aguilar-Roca N. (2009). Garage demos: using physical models to illustrate dynamic aspects of microscopic biological processes. *CBE Life Sci. Educ.* 8 118–122. 10.1187/cbe.09-01-0001 19487500 PMC2689147

[B41] Ruhlman M. (2001). *The Soul of a Chef: The Journey Toward Perfection.* New York, NY: Penguin.

[B42] Runco M. A. Plücker J. Lim W. (2000–2001). Development and psychometric integrity of a measure of ideational behavior. *Creat. Res. J.* 13 393–400. 10.1207/s15326934crj1334_16

[B43] Sohrabi B. Iraj H. (2016). Implementing flipped classroom using digital media: a comparison of two demographically different groups perceptions. *Comput. Hum. Behav.* 60 514–524. 10.1016/j.chb.2016.02.056

[B44] Steen-Utheim A. T. Foldnes N. (2018). A qualitative investigation of student engagement in a flipped classroom. *Teach. High. Educ.* 23 307–324. 10.1080/13562517.2017.1379481

[B45] Strayer J. F. (2007). *The Effects of the Classroom Flip on the Learning end Adornment: A Comparison of Learning Activity in a Traditional Classroom and a Flip Classroom that used an Intelligent Tutoring System.* Doctor Degree Thesis of the Ohio State University, Columbus, Ohio, OH.

[B46] Tang Y. (2005). *Present Situation and Development of the United Kingdom Education Indicators.* Taiwan: Pored publishing company.

[B47] Thai N. T. T. De Wever B. Valcke M. (2017). The impact of a flipped classroom design on learning performance in higher education: looking for the best “blend” of lectures and guiding questions with feedback. *Comput. Educ.* 107 113–126. 10.1016/j.compedu.2017.01.003

[B48] Torrance E. P. (1962). *Guiding Creative Talent.* Englewood Cliffs, N. J: Prentice-Hall.

[B49] Vygotsky L. S. (1987). *The Collected Works of L. S. Vygotsky.* New York, N.Y: Plenum Press.

[B50] Vygotsky L. S. (2004). Imagination and creativity in childhood. *J. Russ. E. Europ. Psychol.* 42 7–97.

[B51] Xu H. C. Huang D. S. Gu D. T. (1996). *Cake and Pastry.* Taipei: China Grain Products Research & Development Institute, 302–324.

[B52] Yeh Y. (2004). The interactive influences of three ecological systems on R & D employees’ technological creativity. *Creat. Res. J.* 16 11–25. 10.1207/s15326934crj1601_2

[B53] Zainuddin Z. (2018). Students’ learning performance and perceived motivation in gamified flipped-class instruction. *Comput. Educ.* 126 75–88. 10.1016/j.compedu.2018.07.003

[B54] Zhang C. X. (2013). *Educational Psychology -Three Theoretical and Practical Orientation.* Taipei: Donghua published.

[B55] Zhong X.-L. Song S.-Q. Jiao L.-Z. (2013). Instructional design based on the idea of the flipped classroom in ICT environment. *Open Educ. Res.* 19 58–64.

